# Virtue Ethics and Integration in Evidence-Based Practice in Psychology

**DOI:** 10.3389/fpsyg.2020.00258

**Published:** 2020-02-18

**Authors:** Henrik Berg

**Affiliations:** Centre for the Study of the Sciences and the Humanities, Faculty of Humanities, University of Bergen, Bergen, Norway

**Keywords:** virtue ethics, clinical expertise, psychotherapy practice, evidence-based practice in psychology, critique

## Abstract

The policy statement for evidence-based practice in psychology is the most important document in contemporary psychotherapy. In its current form, evidence-based practice in psychology gives scientific research precedence in psychotherapy practice. However, psychotherapy practice’s complexity warrants reflection beyond the limits of science. The importance of clinical expert is not recognised in the current policy statement. The clinical expert is necessary to translate psychological research into clinical practice. It is also crucial to identify, clarify and include patient preferences in psychotherapy practice. This paper argues that virtue ethics is a useful theoretical framework for conceptualising clinical expertise. Clinical expertise is conceptualised as the meta-capacity of practical wisdom (*phronesis*) and the virtues necessary for integrating best available research, clinical expertise and patient preferences.

## Introduction

Evidence-based practice in psychology is the most important regulatory policy-statement in current psychotherapy. Its historical background includes the work of Archie Cochrane (Howick, 2011). Cochrane wanted to efface the inferential biases associated with expert-based medicine because they led to ineffective medical practice. According to Cochrane, randomised controlled trials are not susceptible to biases. In randomised controlled trials, researchers test treatment efficacy. Two (or more) equivalent groups are compared. One of the groups are given the treatment, the other is not. The observed differences after treatment are indicators of treatment efficacy. Randomised controlled trials were the building blocks in Cochrane’s vision of a public health-care system (Cochrane, 1999).

The earliest definitions of evidence-based medicine emulated Cochrane’s ideals. Over the course of some decades, however, evidence-based medicine scholars have revised their models several times. A significant change was to expand Cochrane’s model into a tripartite model. In the tripartite model, evidence-based medicine is defined as the integration of best research evidence (preferably, randomised controlled trials), clinical expertise and patient values. In a subsequent revision, Haynes et al. (2002) re-instated the clinical expert at the centre of medical practice. In their model, the clinical expert integrates the three parts in evidence-based medicine.

There is a similar genesis in the regulation of psychotherapy practice. The American Psychological Association launched the criteria for empirically validated treatments (now called research supported psychological treatments) (Chambless et al., 1993). In empirically validated treatments, randomised controlled trials and single-subject designs are used to evaluate psychotherapy schools’ treatment efficacy for various psychiatric disorders. Evidence-based practice in psychology was presented as a more comprehensive alternative to empirically validated treatments. Evidence-based practice in psychology is defined as ‘the integration of the best available research with clinical expertise in the context of patient characteristics, culture and preferences’ (Levant, 2005).

However, Berg has shown that the part ‘best available research’ define and legitimate the two remaining parts ‘clinical expertise’ and ‘patient characteristics, culture and preferences.’ Thus, evidence-based practice in psychology only consists of one part and not of three parts. Nonetheless, there are good reasons for re-defining evidence-based practice in psychology as a tripartite model consisting of best available evidence, clinical expertise and patient characteristics, culture and preferences (Berg, 2019a).

Haynes et al. (2002) argued that a tripartite model needs a clinical expert to integrate the three parts. Psychotherapy researchers have deemed clinical expert as the least developed facet of evidence-based practice in psychology (Norcross et al., 2008). This warrants more conceptual work on the nature of clinical expertise in evidence-based practice in psychology. In this paper, the integrating capacity of the clinical expert is described using virtue theory. The concept denoting the overarching and integrating virtue is *phronesis* or practical wisdom. In addition, there are three classes of virtues corresponding to the three parts: best available research, clinical expertise and patient preferences. These classes of virtues are epistemic virtues, relational virtues, self-reflective virtues. In each class, non-exhaustive examples of specific virtues are given.

### Virtue Ethics and Psychotherapy

Virtue theory originated in Ancient Greek with the works of Plato (2012) and Aristotle (2009). The very definition of virtues indicate why they are relevant for our understanding of clinical expertise. In virtue theory, goodness is defined according to the action’s source. The source of human action is the agent’s character or traits (Waring, 2016). Zagzebski (1996) offers a relatively comprehensive definition of virtue as: ‘[a] multi-track character trait or disposition […] involv[ing] a complex mind-set of fine inner states that inform an array of emotional responses, desires, motivations, reasons and values’ (Waring, 2016).

First, virtues are deep-rooted excellences that exceed mere skill. Like skills, they are cultivated through repetition, but unlike skills they become habitual dispositions which shape the person’s character and outlook on the world (Radden and Sadler, 2010). Virtues, moreover, cause a web of characteristic emotional, cognitive and motivational responses (Waring, 2016). Thus, to have the virtue of compassion is to somewhat automatically feel emotional pain when you see suffering, wanting to understand how to help, activating a set of rationales for why it is important to help and having values consistent with helping suffering fellow human beings (Waring, 2016). Second, virtues enable the person possessing them to bring about certain ends (Pellegrino and Thomasma, 1993). The virtuous psychotherapist is able to produce the end of alleviating psychological suffering and increase life-satisfaction. Third, virtues function to counter therapeutic vices (Pellegrino and Thomasma, 1993). To be compassionate counters being cold or remote, at the one hand and over-involving or invading, at the other.

### *Phronesis* and Integration in Psychotherapy Practice

Virtue ethicists are concerned with questions relevant to contemporary health-care. Aristotle described the type of knowledge and skill most relevant to psychotherapy practice. It is called *phronesis* which can be translated into practical wisdom (Aristotle, 2009). *Phronesis* can be contrasted with two other kinds of knowledge; *sophia* or pure knowledge (e.g., pure mathematics) and *techne* or mere practical skill (e.g., changing a bicycle tire). *Phronesis* is the kind of knowledge and skill that enable human beings to understand and do the right thing in practical matters. It is ‘the link between the intellectual and the moral life […] habitually [disposing us] to attain truth for the sake of action as opposed to truth for its own sake’ (Pellegrino and Thomasma, 1993).

*Phronesis* is acquired through accumulating sufficient practical experience from a variety of situations (including both professional and non-professional experiences) (Selinger and Crease, 2006). These experiences shape our outlook on the world. The manner in which *phronesis* is acquired suggests something about the quality of this kind of knowledge. Unlike mathematics or logic, *phronesis* is not an exact kind of knowledge. It is an approximate knowledge entailing a recognition of:

*‘[…] the anxiety of choice in complex circumstances […] enabl[ing] us to assess the complexities as accurately as possible and to approximate, as closely as the circumstances permit, what would be right and good’ (Pellegrino and Thomasma, 1993)*.

*In clinical practice, phronesis* is the indispensable (Pellegrino and Thomasma, 1993) meta-virtue (Radden and Sadler, 2010). *Phronesis* ‘is a guide to the right way of acting with respect to all the virtues’ (Pellegrino and Thomasma, 1993). In other words, it is the key characteristic for an integrating clinical expert as envisioned in evidence-based practice in psychology. In this context, *phronesis* entails the skill and wisdom necessary for a consistent good practical integration of best available research, clinical expertise and patient characteristics, culture and preferences. Radden and Sadler (2010) have presented an elaborated definition:

*[*Phronesis*] allows us to deliberate about things with ends or goals in mind, and to discern and enact right action. A grasp of particulars is required for *phronesis*, so it comprises cleverness (in the ability to find what is needed to achieve an end or goal), perception (in order to notice facts in a situation) and finally, understanding (*noûs*), a common and practical good sense (12, my italics)*.

Within the evidence-based practice in psychology framework, it means the ability to integrate all of the relevant complexity in clinical action. Evidence-based practice in psychology (implicitly) defines the most relevant complexity through its three parts. These three parts have three corresponding classes of virtues:

(i) Best available research: Epistemic virtues
(ii) Patient culture characteristics and preferences: Relational virtues.
(iii) Clinical expertise: Self-reflexive virtues.

### Epistemic Virtues

A virtuous clinical expert knows what the right means for the right ends are. This includes questions of both fact (means) and value (ends). Moreover, it indicates that theory of knowledge is ethically relevant. Virtue epistemology telescope questions of knowledge with questions of ethical character. The overarching emphasis is the question of being truthful or knowing the truth (Zagzebski, 1996).

There are two prominent traditions in virtue epistemology. These are called reliabilism and responsibilism (Zagzebski, 1996). Reliability argue that ‘[e]pistemic virtues are dependable cognitive tools that enable the inquirer to attain the truth more often than not’ (Waring, 2016). Responsibilists relate epistemic virtues to the ‘intellectual habits and dispositions [and] to the active agency of those who seek truth through inquiry’ (p. 36). Reliability emphasise the outcome and responsiblists emphasise the inquiring agency of the knowing agent. As an overarching ideal, the two positions balance the need for scientific knowledge (reliabilism) with the need to questioning and criticising the knowledge base and its presuppositions (responsibilism). In disciplines with complex objects of inquiry like psychology there are few clear-cut ‘truths’ (Zagzebski, 1996; Waring, 2016). In psychotherapy, the typical ultimate aim is a clinical action that helps the patient. This presupposes a realistic and discerning assessment of the merits and limitations of the knowledge. Thus, integrity and intellectual honesty are good examples of key epistemic virtues (Pellegrino and Thomasma, 1993).

Due to psychotherapy’s complexity, the clinical expert must possess the epistemic virtues to understand the merits of different kinds of knowledge. This entails assessing the epistemic quality in different kinds of knowledge (Guba and Lincoln, 1994; Woolfolk, 2015; Appelbaum et al., 2018; Levitt et al., 2018). The risk of interpretative force-fitting (i.e., to use models inflexibly) makes curiosity and objectivity two relevant epistemic virtues. Furthermore, the clinical expert needs epistemic virtues to understand and sometimes confront the fundamental disciplinary uncertainties in psychotherapy research (e.g., nosology, empirical status, research practices, etc.) (Bohart et al., 1998; Westen et al., 2004; Gupta, 2014; Melchert, 2016; Jackson, 2017) and the entanglement of questions of facts and values in psychotherapy (Woolfolk, 2015; Berg and Slaattelid, 2017). This makes the more holistic concept understanding relevant, as opposed to possessing mere factual knowledge (Woolfolk, 2015).

### Relational Virtues

A classic virtue ethical doctrine states that one should treat only like cases as alike. In psychotherapy, every patient differs in clinically significant matters and, presumably, different patients have different notions of the good life. As noted by Waring (2016), the entanglement of facts and values in psychotherapy entails that ‘[patients] want a normative sense of how the facts and values of their lives “hang together”’ (p. 48). This is therapeutically significant as ‘[i]t can help to organize and reconfigure what patients already know about themselves […] [in] a “larger web of value”’ (Waring, 2016).

The question of what it means for a given patient to live well invoke the patients’ autonomy to decide over her own life. Radden and Sadler (2010) argue that: ‘[p]atient autonomy has become […] the most widely honoured principle in biomedical ethics (p. 114). In normative ethics, autonomy is often tied to the tradition of duty ethics, emphasising the necessity of universal principles and formal rules. Apparently, formal and universal principles contradicts the approximate and “common-sensical”’ nature of *phronesis*. However, in practice the two traditions may be less contradictory. Pellegrino and Thomasma (1993) claim that ‘the virtues are conditions of possibility for the implementation of [such] principles and moral rules’ (p. 28) the power imbalances between therapist and patient is an often-existing obstacle for patient autonomy in psychotherapy. Pellegrino and Thomasma (1993) argue that ‘[t]he [psychotherapist] and the patient are not Lockean free agents equal in bargaining power. The patient is vulnerable […] [and] has not the power to heal herself’ (p. 56). Regulatory principles must encompass the therapeutic relationship and the therapist’s ability to identify and handle obstacles preventing patient autonomy.

In order to obtain patient autonomy, patients must be willing to explore and share their (characteristics, culture and) preferences. This makes trustworthiness a basic relational virtue (Pellegrino and Thomasma, 1993; Radden and Sadler, 2010). Another crucial relational virtue is empathy or compassion. This entails an ability to fathom, and to some extent feel, what the patient is feeling. The demanding emotional nature of psychotherapy makes fortitude another relevant relational virtue (Pellegrino and Thomasma, 1993). The therapist must be perceptive and have the ability to know when to identify, clarify, nuance, challenge, accept and praise a given patient. Patients may have a more or less clear idea about their own culture (or at least the significance of that culture), characteristics and preferences necessary for autonomy. Thus, perceptiveness and discernment must often be combined with patience and perseverance (Radden and Sadler, 2010).

### Self-Reflexive Virtues

Human-beings have the potential for sophisticated self-reflexive thinking and reasoning about our ‘own subjectivity, psychic states and traits, including one’s character’ (Radden and Sadler, 2010). In psychotherapy, the therapist’s interventions reflect various more or less explicit or deliberate moral or ethical stances (Hamilton, 2013; Berg, 2019b). To some extent, this entails that the assessment of a patient reflects the psychological state, personality moral evaluations, knowledge base and socio-cultural background of the therapist. Thus, a hermeneutical reflection of *how* the therapist understands is often as informative as *what* the therapist knows about a patient (Woolfolk, 2015).

Another important argument for promoting self-knowledge is that it creates self-unity. In psychotherapy, self-unity is tied to other therapeutic virtues such as genuineness and wholeheartedness. Psychotherapy consists of a great number of subtle micro-processes (Stern et al., 1998). Self-unity, genuineness and wholeheartedness arguably reduces the probability of detrimental psychic blind-spots influencing the psychotherapeutic work (Radden and Sadler, 2010). This is important to take into consideration because automatic and unconscious content can play a pivotal part in psychotherapy (Maroda, 1991).

Another key self-reflexive virtue is temperance. Pellegrino and Thomasma (1993) argues that temperance ‘represents a kind of victory over desire, a science of the self [and] the individual’s self-mastery is equated […] with wisdom (p. 118). There are many kinds of therapist needs that are impertinent in psychotherapy. These vices lead therapists astray from the ultimate concern in therapy, which is to alleviate the patient’s suffering. One example is therapist idealisation. This makes “unselfing”’ and self-effacement important self-reflexive virtues (Pellegrino and Thomasma, 1993; Radden and Sadler, 2010).

## Conclusion

Emphasising the role of *phronesis* in psychotherapy practice is at odds with the current scientocentric conceptualisation of best psychotherapy practice. The principal contention is whether scientific inquiries have the merits to guide clinical action directly. This paper claims that a good application of scientific results in psychotherapy practice hinges upon *phronesis. Phronesis is* a practical sensibility starting with particulars and reflections regarding what would be a good aim in each individual instance. In addition, psychotherapy practice implies a cluster of facts and values (including patient preferences) necessitating extra-scientific deliberations (Pellegrino and Thomasma, 1993; Berg and Slaattelid, 2017). Virtuous clinical experts are able to integrate the complexities of psychotherapy to the benefit of their patients. This critique does not render scientific findings and patient preferences unimportant. On the contrary, it shows how these elements should be critically evaluated and integrated in clinical practice.

## Author Contributions

The author confirms being the sole contributor of this work and has approved it for publication.

## Conflict of Interest

The authors declare that the research was conducted in the absence of any commercial or financial relationships that could be construed as a potential conflict of interest.
